# Gait Speed improves EuroSCORE II prediction

**DOI:** 10.1186/1749-8090-10-S1-A67

**Published:** 2015-12-16

**Authors:** Felipe B de Salles, Omar AV Mejía, Luiz AF Lisboa, Kalil H Khalil, Luís RP Dallan, Luís AO Dallan, Fábio B Jatene, Marco AP Oliveira, Gustavo I Judas, Sergio A de Oliveira, Orlando Petrucci, Rubens T de Barros, Marcos G Tiveron, Valquíria Campagnucci, Felipe Machado, Amauri Groppo, Rafael Tinelli, Roberto Rocha e Silva, Alfredo Rodrigues, Walter Gomes, Marcelo Nakazone, Domingo Braile, Grupo de Estudo REPLICCAR

**Affiliations:** 1 Department of Cardiovascular Surgery, Heart Institute (InCor), School of Medicine of the University of São Paulo (USP), São Paulo, Brazil; 2 Cardiovascular Surgery, Portuguese Beneficent Hospital of São Paulo, São Paulo, Brazil; 3 Department of Cardiovascular Surgery, Hospital of Clinics of the University of Campinas (UNICAMP), São Paulo, Brazil; 4 Department of Cardiovascular Surgery, Holy House of Mercy of Marília, Marília, Brazil; 5 Department of Cardiovascular Surgery, Holy House of Mercy of São Paulo, São Paulo, Brazil; 6 Department of Cardiovascular Surgery, Holy House of Mercy of Piracicaba, Piracicaba, Brazil; 7 Cardiovascular Surgery, Paulo Sacramento Hospital & SOBAM Group, Jundiaí, Brazil; 8 Department of Cardiovascular Surgery, Hospital of Clinics of Ribeirão Preto, Ribeirão Preto, Brazil; 9 Department of Cardiovascular Surgery, São Paulo Hospital of the Federal University of São Paulo (UNIFESP), São Paulo, Brazil; 10 Department of Cardiovascular Surgery, Hospital de Base of São José do Rio Preto, São José do Rio Preto, Brazil; 11 São Paulo Registry of Cardiovascular Surgery, São Paolo, Brazil

## Background/Introduction

Traditionally cardiac surgery risk scores have worse performance in elderly patients. Frailty evaluation may improve EuroSCORE II accuracy in predicting morbimortality

## Aims/Objectives

Test the value of gait speed, a clinical marker for frailty, to improve the prediction of mortality and major morbidity in elderly patients undergoing cardiac surgery

## Method

A multicenter prospective cohort of patients undergoing coronary artery bypass and/or valve replacement or repair from 12 tertiary care hospitals in São Paulo State/Brazil. Patients were eligible if they were at least 60 years of age. Frailty was defined as slow gait speed, a time taken to walk 5 m of ≥ 6 s. The primary end point was a composite of in-hospital post-operative mortality or major morbidity

## Results

The cohort consisted of 241 patients with a mean age of 67,4 ± 8.2 years. One hundred and two patients (42.3%) were classified as slow walkers before cardiac surgery. Slow gait speed patients were more likely to be female (50.9% vs. 19.4%, p = 0.0001), insulin-dependence diabetic (23.5% vs. 13.6%, p = 0.05), had worse EuroSCORE II (3.9% × 1.8%, p = 0.001). Frail patients had more prolonged length of stay (27.5% vs 7.9%, p = 0.001) and more morbimortality (32.4% vs 15.1%, p = 0.002). Slow gait speed was an independent predictor of the composite end point after adjusting for the EuroSCORE II (odds ratio: 2.36; 95% confidence interval: 1.17 to 4.76) and increased EuroSCORE II accuracy from 67% to 71.9% in prediction of morbimortality

## Discussion/Conclusion

Impaired Gait speed patients have more morbimortality. 5-meter gait speed test is an effective way to identify elderly patients at higher risk and is a simple way to improve EuroSCORE II performance

